# Modeling herbivore functional responses causing boom‐bust dynamics following predator removal

**DOI:** 10.1002/ece3.7185

**Published:** 2021-02-07

**Authors:** Vanessa Haller‐Bull, Michael Bode

**Affiliations:** ^1^ School of Mathematical Sciences Queensland University of Technology Brisbane Qld Australia; ^2^ ACEMS, Australian Research Council Centre of Excellence for Mathematical and Statistical Frontiers Brisbane Qld Australia; ^3^ AIMS@JCU Australian Institute of Marine Science Townsville Qld Australia

**Keywords:** ecological models, functional response, invasive management, invasive species control, Lotka‐Volterra, nonlinearity, predator control

## Abstract

Native biodiversity is threatened by invasive species in many terrestrial and marine systems, and conservation managers have demonstrated successes by responding with eradication or control programs. Although invasive species are often the direct cause of threat to native species, ecosystems can react in unexpected ways to their removal or reduction. Here, we use theoretical models to predict boom‐bust dynamics, where the removal of predatory or competitive pressure from a native herbivore results in oscillatory population dynamics (boom‐bust), which can endanger the native species’ population in the short term. We simulate control activities, applied to multiple theoretical three‐species Lotka‐Volterra ecosystem models consisting of vegetation, a native herbivore, and an invasive predator. Based on these communities, we then develop a predictive tool that—based on relative parameter values—predicts whether control efforts directed at the invasive predator will lead to herbivore release followed by a crash. Further, by investigating the different functional responses, we show that model structure, as well as model parameters, are important determinants of conservation outcomes. Finally, control strategies that can mitigate these negative consequences are identified. Managers working in similar data‐poor ecosystems can use the predictive tool to assess the probability that their system will exhibit boom‐bust dynamics, without knowing exact community parameter values.

## Graphical abstract

Invasive predator control can cause direct negative outcomes if implemented at high levels to populations at risk. This study provides a methodology to identify and hence protect communities at risk.

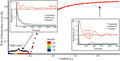

## 1 INTRODUCTION

Invasive predatory species are a primary driver of native species extinction (Doherty et al., 2016; Park, 2004; Vilà et al., 2011). Introduced mammalian predators in particular have caused many extinctions in birds, mammals, and reptiles (Medina et al., 2011; Woinarski et al., 2015), especially on the world's islands. A common conservation response is to control or eradicate problematic invasive species. However, such actions can have unforeseen negative effects for species of conservation concern (Buckley & Han, 2014). For example, the removal of invasive cats *Felis catus* from Little Barrier Island in New Zealand (Rayner et al., 2007) caused a reduction, not an increase, in the breeding success of the threatened Cook's Petrels, because predation by rats *Rattus exulans* increased in the absence of cat predation (a “mesopredator release”). Short‐term low population sizes increase the risk of already threatened species going extinct through loss of genetic diversity, and unpredictable point‐in time threats (e.g., bushfires). Understanding the mechanisms behind unforeseen negative effects help to anticipate and avoid them and is a growing field of conservation theory (Baker et al., 2019; Raymond et al., 2011; Roemer et al., 2002; Zavaleta et al., 2001).

To date, the mechanisms used to explain negative effects following invasive predator control are all based on “indirect effects” (including each of the above examples). In these circumstances, the control of one species A (e.g., cats) to benefit a second species B (e.g., Cook's petrels) fails because an unanticipated interaction occurs via a third species, which is itself often invasive (e.g., rats). Here, we call attention to a more parsimonious, direct explanation for a decline in the native species. In this “direct effects” model, the unanticipated and unintended decline of the native species is partly or entirely endogenous to that species—specifically, the decline occurs as a consequence of removing A too rapidly, and of B’s interaction with its own resources. In the direct effects model, controlling A allows the abundance of B to increase, but this increase overshoots the species’ carrying capacity. After a delay, B will begin to decline due to lack of resources. This overshooting followed by a crash is called boom‐bust dynamics. Populations of B and its resource may continue to cycle, or they may eventually reach a new long‐term equilibrium (which could be higher or lower than the original equilibrium). Even though the long‐term equilibrium might be higher than the original population size, the cycles and especially, the crashes can lead to short‐term population sizes that are lower than the original population size. The problem with bust events is that even short term a low population size can increase the risk of extinction (Pimm et al., 1988).

There are good reasons to suspect that direct effects are responsible for bust events in threatened species conservation. The recommended method of control in Australia is to remove as many animals as possible in a short time period (Department of the Environment & Water Resources, 2007). In other countries like New Zealand, invasive species management is dependent on each area's Council. While about 50% of species are managed (Russell et al., 2015), the management is inconsistent between areas and the control often acts on an high or no control paradigm with intermediate control levels rarely being considered (Brenton‐Rule et al., 2016). However, even with high control intensities only 0.25% of species are eradicated (Russell et al., 2015). Global recommendations by IUCN (McNeely, 2001) are also to apply intense and immediate control. Previous research has noted that this strategy could lead to unintended negative consequences for the species of concern. In Australia, a direct effect model was proposed to explain an increase and crash of bandicoot *Perameles nasuta* populations in Booderee National Park (Dexter et al., 2013; Lindenmayer et al., 2018), and of woylie *Bettongia ogilbyi* populations during the Western Shield fox baiting program in south‐western Australia (Wayne et al., 2011). However, in both cases, herbivore release was only one proposed mechanism for the observed decline, and the specific mechanism and dynamics have not been formally described.

One way to predict community responses to an invasive species or a corresponding management action is modeling of the system. Multi‐species modeling enables the evaluation of different manipulations or management actions of the system in an efficient and no‐risk matter. However, care must be taken with the scope of the model, which species to include or exclude, and the model chosen to represent the system. Many different methods exist for multi‐species modeling, one being a Lotka‐Volterra predator–prey system. Lotka‐Volterra models have been used since 1920 (Lotka, 1920) and since then these models have been commonly used. While the dynamics of the model are not new or surprising, the implication of them to the question of bust events has not been investigated formally. For this kind of system, predator is meant in the widest sense, that is, herbivores and carnivores. Prey refers to the food source, that is, vegetation or another animal.

The problem with ecological models for the purpose of realistic management is the lack of knowledge surrounding population parameters. Many of the parameters cannot be found directly through experimentation so have to be estimated (Abrams, 2001). This can make the model very inaccurate. Only very few parameters such as the intrinsic growth rate of vegetation can be easily measured. Furthermore, the best method to represent species interactions in the mathematical model is not always clear. The simplest form of the predator–prey models is based purely on linear interactions between the predator consumption and the prey density. This linear interaction in ecology is often referred to as a type I functional response. However, even in a simple competition model of *Drosophila*, populations already show nonlinear interactions (Ayala et al., 1973; Gilpin & Ayala, 1973). Other functional responses include type II an asymptotic function and type III an s‐shape function (Holling, 1959a). There is a lot of evidence for these functional response types including experimentations and are quite common in nature but often neglected as soon as several species are introduced (Jeschke et al., 2002). To enable a full exploration of the possible management outcomes, it can be helpful to consider not just linear but several functional response types.

The aim of this study is to investigate the issue of boom‐bust herbivore release in invasive predator control. In particular, we would like to explore and understand the dynamics of these direct, delayed, negative outcomes caused by boom‐bust dynamics; identify communities (through their parameter values) that are likely to display these boom‐bust dynamics; and suggest how these findings could support future management decisions.

## 2 MATERIALS AND METHODS

We approach the problem of herbivore release by first modeling an ecological community that comprises native vegetation, native herbivores, and an invasive predator. We then simulate invasive species management at a range of intensities and observe the population response of both herbivores and vegetation. To ensure that our results are not specific to our choice of model parameters and structure, we investigate the sensitivity of our results to a range of assumptions.

### 2.1 General model with parameter and structural variation

We use a three‐species Lotka‐Volterra system to describe the rates of change in abundance $N_{i}$ of native vegetation (i=1), a native herbivore (i=2), and an invasive predator (i=3):

(1) $$\frac{dN_{i}}{\mathrm{dt}}=N_{i}\left[r_{i}+\sum_{j=1}^{3}A_{\mathrm{ij}}N_{j}\;\text{-}\;c_{i}\right].$$

Although natural systems are more diverse, a three‐species model is simple enough to allow analysis and interpretation, while being sufficiently complex to exhibit the boom‐bust dynamics that cause negative outcomes. Species have intrinsic net growth rates, r (intrinsic growth minus density independent natural mortality), which we assume is zero for the herbivore and predator. Both of these groups only grow by consumption of the lower trophic level. Natural mortality rates of herbivores and predators are negligible in this particular system, since the natural mortality is relatively low compared to either the high predation rate in case of the herbivore or the removal by control in case of the predator.

Each species *j* influences the abundance of the other species *i* via the interaction terms $A_{\mathrm{ij}}$. Control ($c_{i}$) removes a proportion of each population, for example, by baiting, and is zero for all native species.

**FIGURE 1:**
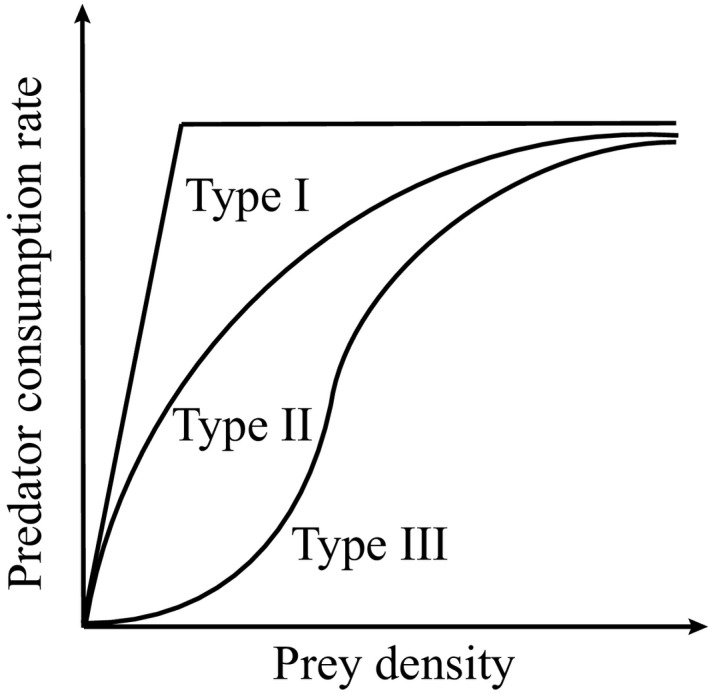
Schematic of the functional response types used in ecology (adapted from Denny, 2014)

The basic implementation of Lotka‐Volterra only considers species interactions as linear functions, known as a “type I” functional response; however, this is often not accurate (Hassell, 1978). Holling (1959b) described three types of functional responses (Figure 1), that is, dependence of predation rate on the prey density (Jeschke et al., 2002). The type II functional response is asymptotic, meaning that the predation rate increases per unit prey density at a declining rate. Holling (1959b) explained this mechanistically with the addition of handling time which is the time necessary for a predator to actually consume the prey. What this means is that once a prey item is caught the predator requires time to kill and consume this prey. This handling time cannot be spent catching more prey, as a consequence, the more prey is caught the more handling time is spent and a limit to how much prey can be consumed is introduced. These dynamics are more realistic than an infinite linear increase in consumption and results in a hump‐shape (Figure 1). Type III functional responses add another feature to the density‐consumption response curve focusing on the interaction at low prey densities. A type III functional response includes a search time as well as a handling time. This search time represents the time required by a predator to find prey. At low prey abundances, it is difficult to find prey so the consumption rate increases slower than linear. As the prey is more abundant, the search time declines but the handling time increases. Both of these dynamics together result in a sigmoidal relationship between prey density and prey capture (Figure 1).

Holling's functional responses can be incorporated into Equation (1) by defining the matrix A as:

(2) $$A=\left(\begin{array}{ccc} ‐a_{11}N_{1}^{n_{1}‐1} & ‐\frac{a_{12}N_{1}^{n_{1}‐1}}{1+a_{12}h_{2}N_{1}^{n_{1}}} & 0\\ \varepsilon_{2}\left[\frac{a_{12}N_{1}^{n_{1}‐1}}{1+a_{12}h_{2}N_{1}^{n_{1}}}\right] & ‐a_{22}N_{2}^{n_{2}‐1} & ‐\frac{a_{23}N_{2}^{n_{2}‐1}}{1+a_{23}h_{3}N_{2}^{n_{2}}}\\ 0 & \varepsilon_{3}\left[\frac{a_{23}N_{2}^{n_{2}‐1}}{1+a_{23}h_{3}N_{2}^{n_{2}}}\right] & ‐a_{33}N_{3}^{n_{2}‐1} \end{array}\right),$$

with $h_{i}$ representing the handling time of species i when consuming species i‐1 it has the unit of tine per number of individuals. $a_{\mathrm{ij}}$ are the interaction coefficients between species i and species j and has the unit of 1 over time. $\varepsilon_{i}$ is the conversion factor between the amount consumed by species i‐1 and the biomass increase of species i, it is unitless. The superscript 1≤n≤3 represents the strength of searching required, that is, the time that a predator needs to first locate a prey item before it can capture it.

**FIGURE A1:**
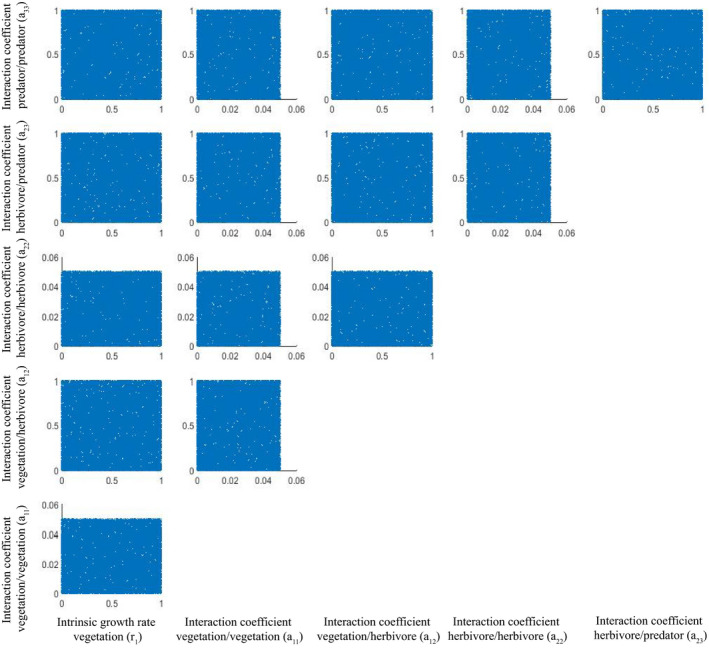
Correlation between the variables in the model

To represent a wide spectrum of communities, we randomly compute parameter sets using a uniform distribution between zero and one for the adjusted Lotka‐Volterra model with $a_{11},a_{12},a_{22},a_{23},a_{33},\varepsilon_{2},\varepsilon_{3}∼U\left[0,1\right]$. This creates theoretical communities of species. The parameters for this model have are all between zero and one, in real communities the growth and interaction parameters can often be larger than one. Hence, to use the results for realistic communities based on collected data, the actual growth ratesn the community have to be scaled to be compared to the theoretical parameter sets used in this study. To scale the data, all parameters need to be divided by the largest value of the parameters resulting in all values being between zero and one. Only communities that have an equilibrium with all $N_{i}>0$ within 20,000 timesteps at a Type I functional response (i.e.,$h_{2},h_{3}=0$ and n=1) are retained for the rest of the computations in this paper. Basically, a system is defined as viable and retained if all populations have a stable, nonzero equilibrium, that is, do not go to extinction. This also means that cycling populations are excluded from the analysis. We acknowledge that this excludes many viable communities; however, the aim of this study is to show introduction of transient cycles and their possible negative effects that can be caused by management interventions. Cycling communities were originally considered for the study; however, preliminary investigation showed that the introduction of a predator control lead to reduced cycles. Consequently, the chance of negative outcomes is low. This combined with the fact that the current analysis relies on equilibria formed the basis for the decision to exclude cycling populations for the purpose of this paper. We acknowledge that this exclusion needs to be taken in consideration when using the results from this study. Furthermore, there was no correlation found between the parameters selected in this fashion (Figure A1).

In this fashion, a total of 1,000 random communities are created. To see the effect that different control intensities have on the community dynamics, the outcome of 10 different control levels $c_{3}∼U\left[0.1,1\right]$ is simulated for each random community and each model structure (Table 1). The model structures represent potentially differing functional response types for the vegetation–herbivore and the herbivore–predator interaction. To investigate the differing outcome for the functional response types II and III thoroughly, we compute 10 levels of handling time $h_{2},h_{3}∼U\left[0,1\right]$ for each of the parameter sets and set n=1 (type II) and n=2 (type III). This means that for each random community we have ~2,400 simulations. This results in a total number of simulations of ~2,400,000 and a resulting sample size of ~2,400,000 outcomes with the associated inputs in the dataset for further analysis.

**TABLE 1:** Parameter choices used to represent the model structures for the three functional response types

Model structures	Vegetation–Herbivore interaction		Herbivore–Predator interaction	
	Type	Parameters	Type	Parameters
1	I	*h*_2_ = 0; *n*_1_ = 1	I	*h*_3_ = 0; *n*_2_ = 1
2	II	*h*_2_ = ~*U*[0,1]; *n*_1_ = 1	I	*h*_3_ = 0; *n*_2_ = 1
3	I	*h*_2_ = 0; *n*_1_ = 1	II	*h*_3_ = ~*U*[0,1]; *n*_2_ = 1
4	II	*h*_2_ = ~*U*[0,1]; *n*_1_ = 1	II	*h*_3_ = ~*U*[0,1]; *n*_2_ = 1
5	III	*h*_2_ = ~*U*[0,1]; *n*_1_ = 1	I	*h*_3_ = 0; *n*_2_ = 1
6	I	*h*_2_ = 0; *n*_1_ = 1	III	*h*_3_ = ~*U*[0,1]; *n*_2_ = 2
7	III	*h*_2_ = ~*U*[0,1]; *n*_1_ = 1	III	*h*_3_ = ~*U*[0,1]; *n*_2_ = 2

### 2.2 Responses

To evaluate the effectiveness and chance of boom‐bust dynamics, we first define the responses of interest. Due to the management interest of the herbivores in this study, we define the response, from now on called “bust event” as the maximum reduction of the herbivore biomass below its original equilibrium (in the absence of control). This reduction could be either permanent or transient. Hence, a bust event could be a short‐term loss of herbivores below the original equilibrium even though the long‐term equilibrium predicts an increase in the herbivore population size. The short‐term reduction is considered here, since it can indicate a risk of extinction. The smaller a population size is at any point in time the higher the risk that an additional threat (e.g., a bush fire) could push this population to extinction. Since the original population sizes differ between random populations, this bust event R is calculated as the maximum proportional reduction in herbivore population size below the original (no control) equilibrium

(3) $$R=\frac{N_{2,0}‐N_{2,\text{lowest point}}}{N_{2,0}}$$

This means that 0≤R≤1 with 1 being the extinction of the herbivore and 0 being no bust event (if there is no decrease in herbivore abundance after the initial release the lowest point is equal to the original (no control) equilibrium).

The second response of concern is the benefit (*B*) of the control action. For this, we utilize the new long‐term equilibrium of herbivores that is possible with the control in place

(4) $$B=\frac{N_{2,\infty }‐N_{2,0}}{N_{2,0}}$$

This means that B<0 indicated a proportional loss of herbivores, B=0 being no benefit and B>0 being the proportional benefit, that is, increase of the herbivore population.

### 2.3 Statistical analysis

The main issue of concern at this point is the potential and size of bust events. To enable some predictions to be made from partial knowledge of the community about the chance of bust events, we conduct a series of bagged tree analyses. Bagged tree analysis is a type of regression used in machine learning. This type of analysis was chosen since it enables classification of the different simulations, creates a predictive model for the response, bust event, and identifies the factors that were important for making the predictions. This could be achieved by a standard regression analysis; however, the response turned out to be very nonlinear, many populations showed no or low risk and some very high risk. This makes a bagged tree analysis more appropriate (Prasad et al., 2006).

We run two bagged tree analyses, a “perfect” analysis using all the information we have and one that represents a more realistic scenario of the data available. First, to get the best (perfect) results we use all of the inputs of the model. This includes all parameters ($r_{1},a_{11},a_{12},a_{22},a_{23},a_{32},\varepsilon_{2},\varepsilon_{3},h_{2},h_{3},n$) plus the level of control ($c_{3}$). While this level of parameter knowledge would be ideal, it would make a statistical model unnecessary, since a mathematical model would be as useful. Second, for more realism we use less parameters and lower certainty about the parameter values. In reality, many parameters are very hard to estimate (Table A1) and even the level of predator control which can be assigned by management actions is usually not known exactly. For example, a target of high fox control efforts might be set, but once implemented effectiveness might only be a removal between 75% and 95% of foxes (Saunders & McLeod, 2007). Therefore, the second bagged tree analysis only uses a subset of the parameters as predictors (Table 2).

**TABLE 2:** Model variables used as predictor subset in the reduced model

Predictor	Explanation	Type	Levels
$h_{2}$	Handling time of population 2 (i.e., herbivores) see Equation (2)	Continuous	[0,1]
$a_{12}$	Interaction parameter see Equation (2)	Continuous	[0,1]
n	Represents the strength of searching see Equation (2)	Categorical	1,2
Control	Proportion of predators removed ($c_{3}$)	Continuous	[0,1]
$r_{1}$	Intrinsic growth of vegetation see Equation (2)	Continuous	[0,1]

This subset of parameters represents those that have the highest chance of being known. One of these parameters is the level of control that is implemented. Since this is a decision made by the management team, it is a known parameter. The other parameters used for the subset are the intrinsic growth rate of the vegetation and the parameters representing the functional response between herbivore and vegetation. The former can be found through an exclusion experiment, raise the vegetation in absence of any herbivory. The latter through an experiment that varies vegetation density and herbivore abundance with the aim to measure consumption rates and fit the response curve (Haddaway et al., 2012; Holling, 1959a). This enables us to investigate the degree to which we could still determine a solution in a more realistic management scenario.

However, even with an estimation of the parameters they are never fully known. Consequently, to include parameter uncertainty into the bagged tree analysis, we vary the input parameters within ±10% of the original to mimic the uncertainty that would exist around any parameters estimated from real world data. For each sample and variable in the dataset, we compute a random variable between 0.9 and 1.1 and multiply it with the original datapoint. This creates a new dataset that includes variation. For the training data, this is done once. For the validation data, this is repeated 100 times and each of these datasets is then used to predict the chance of a bust event (*R*); in the results, we present the average plus standard error of these repeated predictions.

To enable easier classification within the bagged tree analysis, the response variable *R* was divided up into five categories very low bust event 0≤R<0.2, low bust event 0.2≤R<0.4, medium bust event 0.4≤R<0.6, high bust event 0.6≤R<0.8, and very high bust event 0.8≤R≤1.

## 3 RESULTS

### 3.1 Can bust events be intrinsic to the system?

**FIGURE 2:**
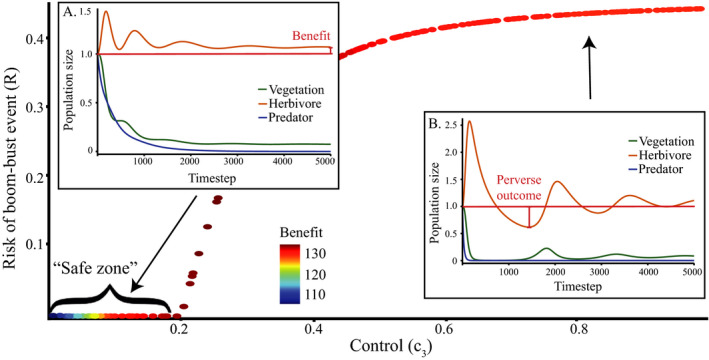
Bust event (*y*‐axis) and benefit (marker color) as a function of increasing invasive predator control intensity (*x*‐axis), for the standard Lotka‐Volterra and one set of parameters (***r***_1_ = 0.29, ***a***_11_ = 0.02, ***a***_12_ = 0.19, ***k***_2_ ***a***_12_ = 0.37, ***a***_22_ = 0.02, ***a***_23_ = 0.48, ***k***_3_ ***a***_23_ = 0.04, ***a***_33_ = 0.09; ***r***_2_, ***r***_3_, ***a***_13_, ***a***_31_, ***h***_2_, ***h***_3_ = 0; ***n***_2_, ***n***_3_ = 1). The “safe zone” is a range of control levels $c_{3}<0.38$ that incur no bust event to the native herbivore population. Inset panels show the abundance of the three species through time following the application of control. a: low control $\left(c_{3}=0.1\right)$; b: high control $\left(c_{3}=0.8\right)$

Invasive predator control pushes the ecosystem toward a new equilibrium, sometimes with transient oscillatory dynamics. Lower control intensities often cause a monotonic increase in the herbivore population, with no herbivore release (Figure 2a); we call this a “safe zone” for the control parameter. At higher control intensities, however, oscillations emerge, and increase in magnitude with the control parameter. These oscillations can push the herbivores’ population below its original equilibrium, and cause a bust event (Figure 2b). Interestingly, benefit does not continuously increase with increasing control intensity (Figure 2). While safe zone and maximum benefit can be found in most communities, the exact control intensity associated with each characteristic is dependent on the population parameters sets.

### 3.2 Can we predict bust events? What is important for the predictions?

Next, we look at all three functional types in the two bagged tree analysis to determine what parameter value combinations are associated with bust events. The first result to note here is that we can create a model that can predict bust events with high accuracy when considering all predictors (Table 2). However, more interestingly, the second and more realistic bagged tree analysis found reasonable accuracy when only using a few inputs and variations (Table 3), that is, the control level, the intrinsic growth of vegetation and the parameters that describe the interaction between herbivores and vegetation ($a_{12},h_{2},n_{2}$). The major decrease in accuracy between fitting a model on the full (first analysis) or partial parameter set (second analysis) is found when predicting the medium bust event groups, however, these also reflect the least number of data points. Although the lowest chance of bust event group is predicted with on average 69% accuracy (Table 4). This is much lower than with the full model (Table 3); however, the accuracy of the high bust event group remains above 96% (Table 4).

**TABLE 3:** Confusion matrix for the bagged tree analysis on a validation dataset fitted with all model parameters as predictors

	Predicted bust event				
	Very low	Low	Medium	High	Very high
Measured bust event					
Very low	96.8	0.1	0	0	3.1
Low	0.2	99	0.1	0.1	0.6
Medium	0	0.3	92	0.4	7.3
High	0	0	0	86	14
Very high	0	0	0	0	100

Note

Each element in the table reports the percentage of measured outcome that was classified at each level of the predicted outcome. Overestimated bust event classifications are highlighted in yellow and underestimated bust event classifications are highlighted in red.

**TABLE 4:** Confusion matrix for the bagged tree analysis on a validation dataset fitted with the subset of model variables as predictors (Table 2)

		Predicted bust event				
		very low	Low	medium	high	very high
Measured bust event	Very low	68±0.00	0.86±0.00	0.08±0.00	0.05±0.00	30.28±0.00
	Low	38.85±0.06	45.23±0.05	0.37±0.01	0.27±0.01	15.28±0.05
	Medium	23.25±0.18	6.23±0.10	40.75±0.21	0.20±0.03	29.57±0.21
	High	38.05±0.39	7.06±0.27	0.11±0.03	40.94±0.30	13.84±0.10
	Very high	3.05±0.00	0.12±0.00	0.04±0.00	0.00±0.00	96.79±0.01

Note

Each element in the table reports the percentage of measured outcome that was classified at each level of the predicted outcome. The percentages also include an estimate of the standard error based on the differences of 10% parameter variation. Overestimated bust event classifications are highlighted in yellow and underestimated bust event classifications are highlighted in red.

**FIGURE 3:**
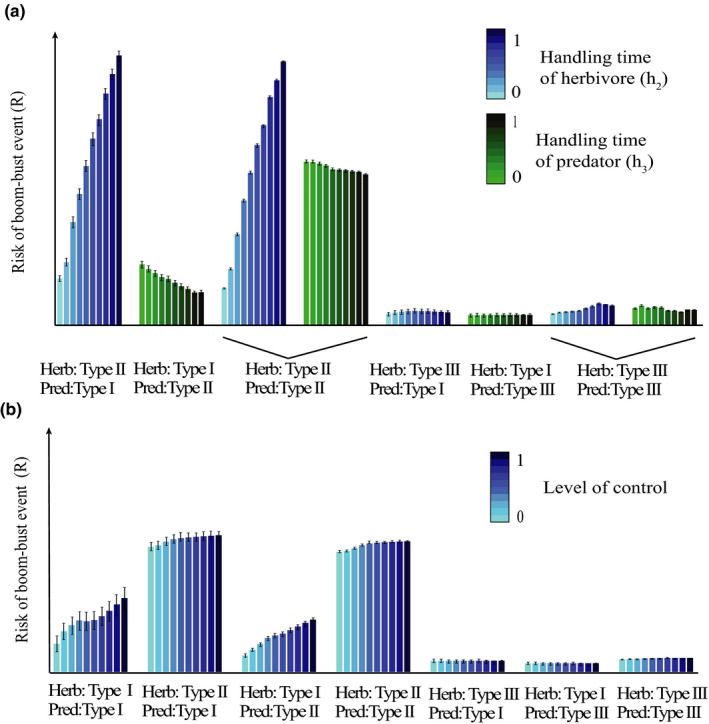
Average (±*SE*) of bust events split up according to models and variables. a. Shows the influence that the handling time (*h_2_* or *h*_3_) has on the bust event split for the models that include type II and type III functional responses. The models are named according to the functional responses included, for example, Herb: Type II, Pred: Type I refers to the model that sets the vegetation–herbivore interaction as Type I (*h*_2_ = 0 and *n*_2_ = 1) while the herbivore–predator interaction is Type II (*h*_3_ = [0,1], *n*_3_ = 1). b. Shows the influence of the control level on the bust event based on all types of models investigated. Model specification is the same as in part a

Next, we show how some of these important predictors influence the bust event. The handling time of herbivores (*h*_2_) impacts the bust event more than the handling time of predators (*h*_3_) (Figure 3a), which makes sense since it is the relationship between herbivores and vegetation that is responsible for a herbivore release. Bust events are lower with type III functional responses at both the herbivore and the predator level. Accordingly, the amount of influence that the level of control has on the bust event is also mostly dependent on the functional type of the vegetation–herbivore interaction (Figure 3b). A higher control usually increases the bust event; however, this is most pronounced when the vegetation–herbivore interaction is of functional response Type I. Between the response types, it is clear that type II responses of herbivores show the highest overall bust events, followed by type I responses of herbivores. Finally, the lowest bust event is found when a functional response type III is involved in the system.

## 4 DISCUSSION

Our results predict and explain the existence of a boom‐bust herbivore release phenomenon, when managing threatening predatory invasive species. The magnitude of this herbivore release is related to the intensity of control actions. It can be avoided by lower control efforts, but at the cost of long‐term benefits for native species. Its existence and magnitude may be predictable, to some extent, with a limited understanding of the system parameters.

### 4.1 Direct cause for bust events common

Strong empirical evidence indicates that invasive predator control actions create an immediate, direct benefit for threatened native species (Dexter et al., 2013; Kinnear et al., 2002). This is reflected in our models. However, these same models predict that this initial benefit can transform into a delayed, transient, bust event for species of conservation interest in the medium‐term. Paradoxically, it is the magnitude and speed of the initial benefit that creates the delayed bust event. An example of this is shown in Figure 2a, at high control intensity (0.8) the initial increase in herbivore abundance is so high that it triggers a decline in vegetation. In general, intense control causes rapid declines in predator numbers, which lead to the fastest increase in herbivore abundance; when this causes an over‐consumption of vegetation, the herbivore population crash becomes likely. The safe zone of low control intensity avoids this outcome by reducing the size of these oscillations, but once control intensity exceeds the safe threshold, a trade‐off between bust event and benefit occurs. Specifically, higher levels of control increase the chance of short‐term extinction, but also increase equilibrial populations. These results highlight that direct effects can cause the bust events without any need to leap to indirect explanations. However, this does not mean that indirect mechanisms, such as an alternative predator entering the area, should not be considered. Thorough research into teasing apart how much of the decline is caused by each explanation should be conducted to avoid extinctions.

### 4.2 Predictions are possible even with reduced information

While it would be ideal to measure all of the parameters, and then use the resulting bust event–benefit relationship to inform management action, accurately estimating parameters in real ecosystems is essentially impossible (Abrams, 2001; McCallum, 2008). Fortunately, our analyses suggest that a few key parameters are sufficient to provide insight into the level of bust event. Especially the high bust event groups can still be predicted well which can lead to conservative management choices. For example, the intrinsic growth rate of vegetation could be estimated in ex situ experiments, while the Hollings functional type and interaction strengths can be estimated by in situ manipulation of densities, and observation of consumption rates (Haddaway et al., 2012; Holling, 1959a). Haddaway et al. (2012) conducted this type of experiment on invasive crayfish *Austropotamobius pallipes* and their prey species. They exposed the crayfish to 14 different prey densities of 4–320 individuals per tank. Then they fitted a functional response type II. In the system, we are considering here, this kind of experiment is only possible for the herbivore as consumer and the vegetation as consumed due to animal ethics (Garner, 2005; Rollin, 2006).

The bagged tree analysis for the parameter subset has reasonable accuracy even though it is lower than the analysis based on the full parameter set. The strength of the bagged tree analysis is that it can cope with the missing information in form of missing parameters. The bagged tree analysis on the subset produces higher errors than with the full set of information; however, the analysis allows for the error to target specific results. For example, the results presented in this paper minimized the error for high chance of bust event groups, while allowing for higher error in lower chance of bust event groups. This was done to provide a conservative look at management actions, that is, a managers might be more interested in correctly identifying high risk actions. If the manager is concerned with another feature, then the analysis can be adjusted to minimize those errors. While this analysis is not without misestimation of outcome, it still provides useful insight and cautions.

The overall accuracy of the predicted outcomes even with less information can be explained when considering the average bust event for the different levels of the population parameters. Most change in the bust event are a consequence of changes in the functional response type; within each different functional response type, the handling time of herbivores has the largest influence on the size of the bust event. The functional response types exhibit a clear hierarchy, with type II causing the highest bust event, followed by type I and type III producing the lowest bust event. However, this only remains true if the type II response is located at the vegetation–herbivore interaction. Interestingly, a type III response always led to a lower bust event independent of its location in the vegetation–herbivore or herbivore–predator interaction.

### 4.3 Causes for bust events can be found in the math

This hierarchy of bust event by functional response types can be explained by considering the shape of the curves. The two main causes of the magnitude of bust event is first the height of the herbivore release caused by the herbivore growth rate and the reaction time of herbivores to the vegetation depletion and secondly the recovery of the vegetation as well as the reaction time of herbivores to the recovery of the vegetation., that is, how soon the herbivore population crashes, and how low the herbivore population crashes which is determined by the herbivore consumption rate at different vegetation densities.

**FIGURE 4:**
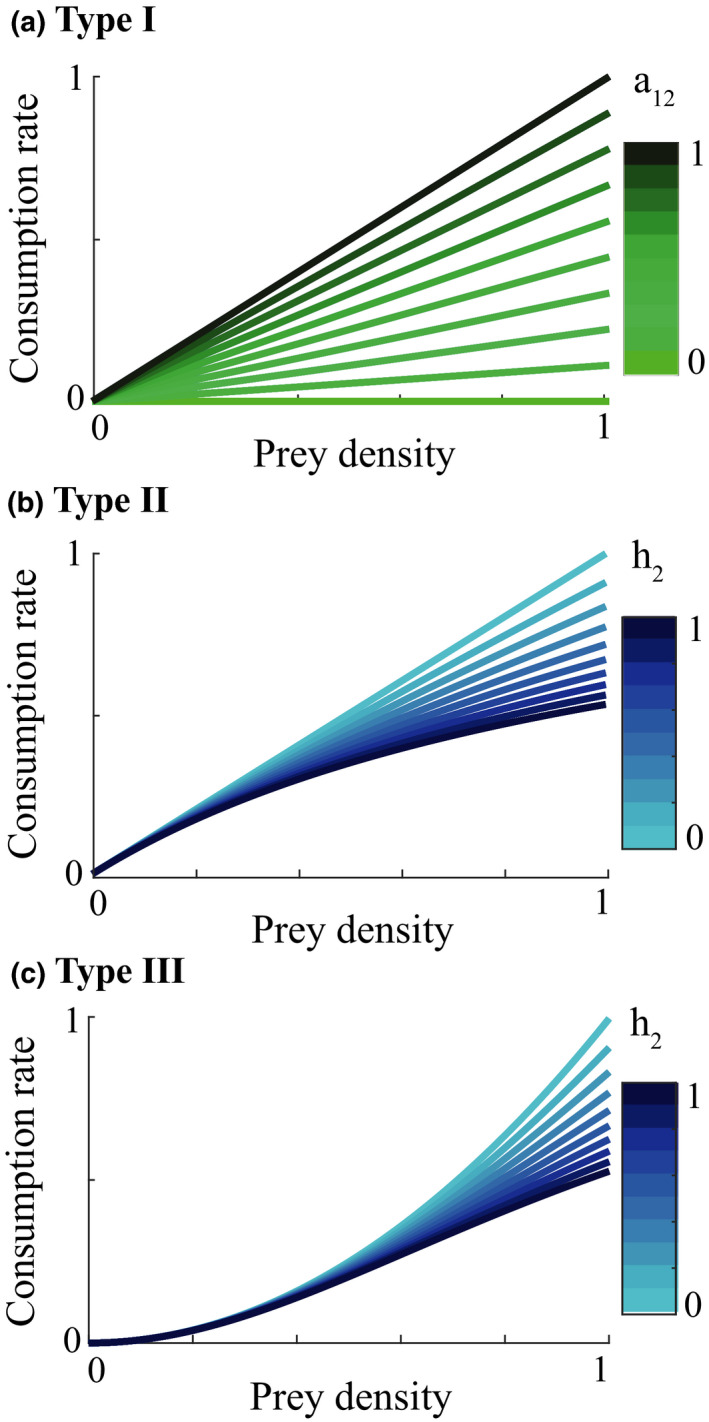
Functional response curves for type I to III while varying the parameters. For section a: we vary $a_{12}$ ($h_{2}=0$). For section b and c: $a_{12}=0.9$ and varying $h_{2}$

When investigating the relationship between prey density and predator consumption that underlies the functional responses, we can see that we have different concavity with the different responses. We assume that at the start of the control measure, the herbivore population is low in density (otherwise we would not need a control of predators). Once the predator is removed and herbivore densities increase, their consumption of vegetation increases which leads to a decrease in the density of vegetation Type I functional response of course is linear (Figure 4a). Type II is concave (Figure 4b) and type III convex (Figure 4c). The main influencer on the level of concavity within each functional response is the length of handling time with an increase in handling time from 0 to 1 increasing the concavity/convexity respectively.

When the type I functional response occurs the consumption rate of herbivores is consistently adjusted as the vegetation density decreases. This leads to a less erratic response and dampened over‐and undershooting. On the other hand, the nonlinearity of type II and III causes more erratic growth patterns but also differing reaction times at low and high vegetation densities. With both type II and type III functional responses, the herbivore consumption rate remains high until it plummets quickly which is a characteristic of its concavity. This allows the herbivore population to continue to grow, even as the vegetation density begins to decrease. The collapse of vegetation, followed by a collapse of the herbivore population, then becomes more likely.

The second characteristic—the depth of the crash—is determined by the behavior of the consumption rate at low vegetation densities. With a type II functional response, the fast decrease of herbivore consumption rate occurs at very low vegetation densities. On the other hand, with a type III functional response the consumption rate plummets at slightly higher vegetation densities and settles very close to zero at low vegetation densities caused by the switch to convexity. This means that the vegetation can start recovering at higher herbivore densities than with a type II functional response resulting in a lower crash for the herbivore population.

The handling time differentiates between type I and type II functional responses. At low handling times, the functional responses become more similar, and the type I response is a limiting case of the type II response, when the handling time is zero. This relationship between response types I and II is also clear when considering the influence of handling time on the bust event. The bust event becomes higher when we have a larger handling time, that is, when the functional response curve is less linear and more concave. On the other hand, when we have a functional response of type III, a higher handling time leads to smaller degree of convexity of the curve which could lead to an increase in bust event. Overall, the influence of handling time in a type III functional response on the bust event is relatively large compared to functional response type II.

### 4.4 Management recommendations

Designing a management strategy for an invasive species is a complex issue with many considerations such as feasibility, economics as well as ecology. One major component of the management strategy is the control intensity that is deployed. In contrast to fundamental population parameters, the control level is anthropogenic and can therefore be directly changed. Invasive species control protocols often recommend that in a short time period as many animals as possible are removed; our results suggest that this might not always be the best strategy. However, this study shows that depending on the community around the invasive species intense control can lead to boom‐bust dynamics and a short‐term decline in the herbivore population. Hence, we propose that one of the first ecological consideration should be if there is a high risk of boom‐bust dynamics. The assessment method that should be chosen to evaluate the risk of boom‐bust dynamics will depend on the amount of data available for the species. If parameters for all species and interactions are known, then the method is simple, structure a population model, and simulate the community's response. This situation is very rare; hence, the predictive tool can be used instead of a full population model.

To use the predictive tool for a community the following steps need to be taken. First, the essential information which are rough estimates of the growth rate of the vegetation and the functional response parameters for the vegetation–herbivore interaction needs to be collected. This could be done by identifying studies of similar species in the literature or designing your own experiments growing vegetation in the presence and absence of herbivores. Second, the data need to be scaled between zero and one to fit within the ranges of the communities investigated in this study. This can be done by identifying the largest of the parameters and dividing all other parameters by this number. Third, the predictive model can be run on the new sample, which is the scaled parameter sets. Ideally, some variation for the parameter sets should be included by varying each parameter by a certain percentage. The percentage can be based on the source of the data, for example, data based on previous studies on similar habitats might be less certain, since the species of vegetation could differ, than data based on experimentation designed specifically for this purpose. The predictive tool will then provide a percentage estimate of the chance that the community could experience boom‐bust dynamics.

Based on the output from the predictive tool, the researcher can then give recommendations on how carefully the control strategy needs to take into account boom‐bust events.

### 4.5 Limitations

There are two main limitations to the application of the results from this study. First, the study does not include any considerations besides the direct effects causing bust events of invasive herbivores. The trade‐offs that can result from the alternative considerations to management can alter the recommendations in terms of control intensity provided in this study quantitatively and qualitatively.

Second, the study is based on a three‐species ecosystem model; in reality, ecosystems have many more species, trophic levels, stochasticity, and spatial structure. This study does not consider competition, internal population structures, or behavioral responses. More complex systems models would be needed to confirm if these phenomena will still occur. Furthermore, the study considers that populations are at an equilibrium at the start of the predator control. This would only be a reasonable assumption when the invasive species is well‐established and not recently introduced. An application to a real ecosystem where this phenomena has been suspected would be a valuable extension of this theory.

Both of these limitations need to be considered carefully when using the results from this study; however, the study provides a foundation to show direct bust events and provides a workflow that could be used with more complex models in variety of settings. Hence, both limitations also represent opportunities for further investigation.

### 4.6 Conclusions

In conclusion, even with a simple three‐species system, we can already have direct species interactions that lead to a delayed collapse of the species of conservation concern. Our methods suggest a novel approach to assess whether such bust event could occur, but they also highlight the importance that model structure (here nonlinear species interactions) can have on the outcome of a study. It reminds us that we need to design the right model to assess the question and that complexities (nonlinearity) should not be excluded, unless an assessment of its impact on the question in mind has been made. Overall, there are two main points to take away from this study: Firstly, managers or researchers directly informing management of invasive species need to consider the functional response types of their lower trophic levels to enable the best choice of control. Secondly, the control level of the invasive species control can influence the chance of bust events and should be carefully considered based on scientific evidence.

## CONFLICT OF INTEREST

The authors declare that there is no conflict of interest.

## AUTHOR CONTRIBUTIONS

**Vanessa Haller‐Bull:** Conceptualization (equal); formal analysis (lead); investigation (lead); methodology (lead); project administration (lead); resources (lead); software (lead); writing – original draft (lead); writing – review and editing (lead). **Michael Bode:** Conceptualization (equal); formal analysis (supporting); investigation (supporting); methodology (supporting); project administration (supporting); supervision (lead); writing – original draft (supporting); writing – review and editing (supporting).

## Data Availability

There were no data used for this study.

## APPENDIX 1

### EXPLANATION OF PARAMETERS IN THE MODEL

**TABLE A1:** Description of the parameters in the model and how/if they can be estimated

Parameter	Description	Estimate possible?
$r_{1}$	Intrinsic growth rate of vegetation	Can be estimated by growing vegetation in absence of any herbivory
$c_{3}$	Rate of removal (control) of predators	Can be set
$a_{11}$	Intraspecific competition within vegetation	Difficult/impossible to estimate
$a_{12}$	Interaction term between vegetation and herbivores	The curve for herbivore consumption rate versus vegetation density can be approximated by an experiment (see below) which gives us $a_{12}$,$h_{2}$,$k_{2}$
$a_{22}$	Intraspecific competition within herbivores	Difficult/impossible to estimate
$a_{23}$	Interaction term between herbivores and predators	The experiment for the consumption curve is not feasible for high value species due to ethics
$a_{33}$	Intraspecific competition within predators	Difficult/impossible to estimate
$h_{2}$	Handling time that herbivores take to consume vegetation	The curve for herbivore consumption rate versus vegetation density can be approximated by an experiment (see below) which gives us $a_{12}$,$h_{2}$,$k_{2}$
$h_{3}$	Handling time that predators take to consume herbivores	The experiment for the consumption curve is not feasible for high value species due to ethics
$k_{2}$	Fraction of the amount of vegetation biomass removed that is passed onto the next trophic level, that is, the fraction that results in herbivore growth	The curve for herbivore consumption rate versus vegetation density can be approximated by an experiment (see below) which gives us $a_{12}$,$h_{2}$,$k_{2}$
$k_{3}$	Fraction of the amount of herbivore biomass removed that is passed onto the next trophic level, that is, the fraction that results in predator growth	The experiment for the consumption curve is not feasible for high value species due to ethics
n	Strength of searching required, that is, the time that a predator needs to first locate a prey item before it can capture it	Can be estimated for lower trophic levels with experiment for predator consumption rate
$N_{1}$	Vegetation biomass	Can be estimated from observational studies
$N_{2}$	Herbivore biomass	Can be estimated from observational studies
$N_{3}$	Predator biomass	Can be estimated from observational studies

Note

The main experiment suggested to fit a functional response curve is as follows. The prey is kept in enclosures at different densities then a predator is introduced (Akre & Johnson, 1979; Haddaway et al., 2012). Over time, we can then record the consumption of this predator for each of the enclosures. With enough replication, we should be able to fit a first second‐ or third‐order polynomial to the data points which would then enable us to estimate the parameters (*a*,*h*,*n*).
